# Small‐Vessel Disease in the Heart and Brain: Current Knowledge, Unmet Therapeutic Need, and Future Directions

**DOI:** 10.1161/JAHA.118.011104

**Published:** 2019-02-02

**Authors:** Colin Berry, Novalia Sidik, Anthony C. Pereira, Thomas J. Ford, Rhian M. Touyz, Juan‐Carlos Kaski, Atticus H. Hainsworth

**Affiliations:** ^1^ British Heart Foundation Glasgow Cardiovascular Research Centre Institute of Cardiovascular and Medical Sciences University of Glasgow United Kingdom; ^2^ The Queen Elizabeth Hospital Discipline of Medicine University of Adelaide Central Adelaide Local Health Network Coronary Vasomotion Disorders International Study Group (COVADIS) Adelaide Australia; ^3^ Department of Neurology St George's University Hospitals NHS Foundation Trust London United Kingdom; ^4^ Department of Cardiology St George's University Hospitals NHS Foundation Trust London United Kingdom; ^5^ Faculty of Medicine UNSW University of New South Wales Sydney NSW Australia; ^6^ Molecular and Clinical Sciences Research Institute St Georges University of London United Kingdom

**Keywords:** angina, cerebrovascular disease, endothelin‐1, magnetic resonance imaging, microvascular dysfunction

Ischemic heart disease (IHD), stroke, and dementia are leading causes of death and disability worldwide,^1^, ^2^ notably affecting aging populations. The public health burden related to chest pain is substantial and the epidemiology of IHD because of large‐vessel coronary atherosclerosis is well documented.^2^ By contrast, the epidemiology of small‐vessel disease (SVD) in the heart is less well established.^3^, ^4^ Cohort studies indicate that the underlying cause of anginal chest pain may be SVD in more than 1 in 3 of all‐comers with stable symptoms.^3^, ^4^ IHD because of SVD associates with vascular risk factors, such as hypertension and female sex.^3^, ^4^, ^5^, ^6^

The vascular anatomy of the heart and brain is similar in that conduit arteries are distributed on the surface of these organs with tissue perfusion achieved through deep penetrating arteries. In the heart, SVD involves the deep penetrating coronary arterioles and the subendocardial plexus of microvessels.^7^ The clinical sequelae of SVD in the heart include stable and acute coronary syndromes and heart failure in the longer term.^3^, ^4^ SVD in the brain mainly involves small subcortical cerebral arteries. Occlusion of 1 of these vessels may result in a clinical stroke syndrome known as a lacunar syndrome. Acute imaging may show a lesion (<20 mm) on diffusion‐weighted magnetic resonance imaging (MRI) indicating an acute lacunar infarct. Later imaging may continue to identify the resulting end‐stage lesion as a lacune (<15 mm). Long‐term ischemia from SVD may show only white matter hyperintensities with or without lacunes and may manifest as vascular cognitive impairment.^8^, ^9^ SVD may manifest as a multisystem disorder^10^ implying commonality between disorders of small vessels of the heart and brain (and potentially other organs such as the kidney) (Figure 1).

**Figure 1:**
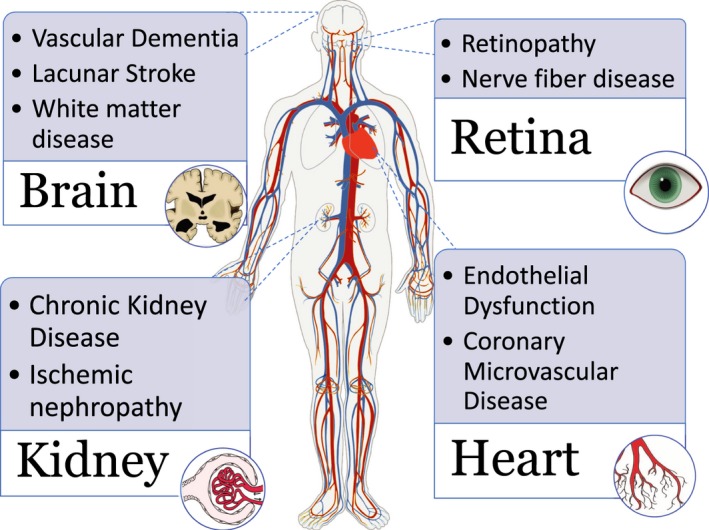
Microvascular disease as a multisystem disorder.

In this article, we review the co‐existence of SVD in heart and brain. We consider evidence for and against a pathophysiological link between SVD in the heart and brain. We identify gaps in knowledge and disease‐modifying therapy. Clinical cases are presented in Figure 2.

**Figure 2:**
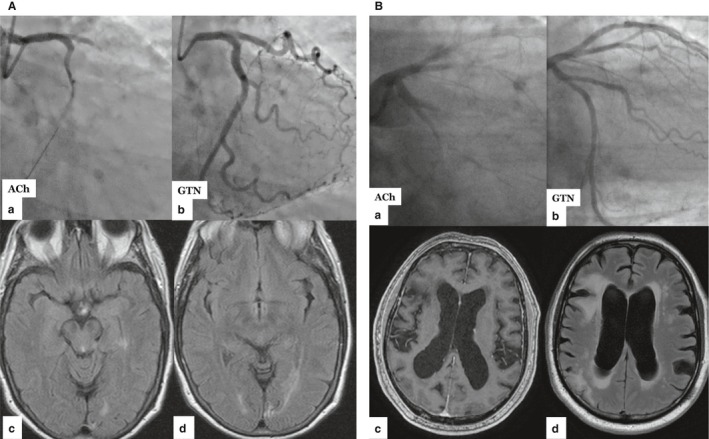
Two clinical cases of patients with microvascular angina who experienced an acute ischemic stroke within 12 months of diagnosis. **A**, A 69‐year‐old woman with background of hypertension and treated dyslipidemia underwent invasive angiography for the investigation of typical angina. She was enrolled in the CorMicA clinical trial (ClinicalTrials.gov Identifier: NCT03193294). Her coronary angiogram was normal and as per the trial protocol she underwent blinded assessment of coronary artery function. Endothelial function was grossly abnormal using an acetylcholine probe (10^‐6^ ‐ 10^‐4^ mol/L infused for 2 minutes). **A**, During acetylcholine, the patient has transient loss of flow in the left coronary artery despite no gross epicardial coronary diameter change. This represents intense microvascular vasoconstriction with absence of contrast in the lumen. There were associated dynamic ST‐segment changes on ECG with reproduction of angina. **B**, After GTN the flow returns to normal with prompt ECG and symptom resolution. Six months later she presented with generalized headache and bilateral visual disturbance and was found to have a right homonymous hemianopia. **C** and **D**, The MRI brain scan shows a left posterior circulation infarct involving the temporal and occipital lobes. **B**, A 67‐year‐old man underwent invasive coronary angiography for severe angina (CCS IV). His background history included myocardial infarction with nonobstructive coronary disease (MINOCA), hypertension, paroxysmal atrial fibrillation with previous stroke, stage III chronic kidney disease, obesity, and moderate left ventricular impairment. Invasive coronary angiography showed nonobstructive coronary disease confirmed with pressure wire (yellow arrow) physiological assessment of the left anterior descending artery (LAD fractional flow reserve 0.84). Indices of coronary microvascular function using adenosine as an endothelial independent probe were profoundly abnormal. The index of microvascular resistance measured in the LAD coronary artery was 49 (abnormal >25) and the coronary flow reserve in the same artery was 1.7 (abnormal < 2.0). Endothelial function testing with acetylcholine provoked slow flow (Thrombolysis in Myocardial Infarction (TIMI) grade 0) (**A**), which represents intense inappropriate microvascular constriction during 10^‐4^ mol/L acetylcholine infusion. Reproduction of angina and ECG changes ensued in keeping with microvascular spasm–induced ischemia. Changes promptly resolved with GTN (**B**). An MRI brain (**C**) scan is shown after his previous stroke, which was attributed to atrial fibrillation. The scan shows no evidence of intracranial mass lesions, abnormal enhancement, or signs of raised intracranial pressure. There is marked dilatation of the lateral and third ventricles with right frontal and right parietal cortical malacia and underlying gliosis in keeping with infarcts. The FLAIR sequence (**D**) shows periventricular white matter changes and multifocal punctate white matter hyperintensities that are typical of SVD affecting the brain. CCS indicates Canadian Cardiovascular Society; GTN, Glyceryl Trinitrate; MINOCA, Myocardial Infarction with No Obstructive Coronary Artery disease; MRI, magnetic resonance imaging.

## Methods

We undertook a literature search for original research articles including information on SVD in both heart and brain. The search used PubMed and covered the period January 1, 1973 to May 31, 2018. We searched for Human studies in English that included these terms in the Title or Abstract (small‐vessel disease, microvascular, arteriolar, arteriole, arteriolosclerosis, leukoaraiosis) AND (heart, cardiac, cardiol, myocardial, myocardium, angina, Syndrome X) AND (Brain, cerebral, cerebrovascular). This search yielded 513 hits and 2 researchers (N.S., A.C.P.) independently screened the abstracts. Eighteen abstracts were selected and the outputs were discussed by 4 investigators (N.S., A.C.P., C.B., and A.H.H.). By consensus, we identified 9 research articles that provided information on SVD in both the heart and the brain, and 1 other on microvascular disease in the kidney and brain. The search was updated on October 12, 2018 and no new original articles fulfilling these criteria were identified. The totality of evidence was insufficient to support a meta‐analysis. The articles that are included in this review^11^, ^12^, ^13^, ^14^, ^15^, ^16^, ^17^, ^18^, ^19^ are summarized in Table 1. The articles that are not included are summarized in Table S1.

**Table 1:** Original Research Articles Describing SVD in the Heart and Brain, and 1 Other on SVD in the Kidney and Brain

Authors/Y	Organ	Design	Objective	Focus	n	Key Findings
Brunelli et al 1996^11^	Brain & heart SVD	Descriptive study	Measure the cerebral blood flow and cerebrovascular vasodilator reserve in patients with coronary microvascular dysfunction and in controls	Patients with coronary microvascular dysfunction	16	Cerebral blood flow and cerebrovascular vasodilator reserve were preserved in a series of patients with coronary microvascular dysfunction, which is not consistent with the hypothesis of a diffuse smooth‐muscle disorder
Sun et al 2001^12^	Brain & heart SVD	Case–control study	Investigate whether coronary microvascular dysfunction is a systemic vascular disorder	Patients with coronary microvascular dysfunction	40	23/25 cases with definite myocardial perfusion defects diagnosed by thallium‐201 myocardial perfusion SPECT also had multiple hypoperfusion areas in the brain vs 2/15 patients without thallium myocardial defects. The parietal lobes were the most common hypoperfusion areas, and cerebellum was the least common
Lesnik Oberstein et al 2003^13^	Brain & heart SVD	Descriptive study	Determine whether myocardial ischemia is associated with NOTCH3 mutations	Members of 15 unrelated families with CADASIL	63	NOTCH3 mutations associated with myocardial ischemia (10 patients with evidence of MI—5 silent); MI predates neurological symptoms (5 patients with MI) and coronary angiography (4 patients) showed unobstructed coronaries; 1 pathology study—myocardial tissue showed no macroscopic stenosis but abnormal microvasculature
Pai et al 2003^14^	Brain & heart SVD	Case–control study	Investigate whether coronary microvascular dysfunction is a systemic vascular disorder	Patients with coronary microvascular dysfunction	30	Coronary microvascular dysfunction is a systemic vascular disorder with a high incidence of hypoperfusion lesions of the brain based on the findings of Tc‐99m ECD brain SPECT, and is usually coincident with myocardial defects based on the Tl‐201 myocardial perfusion SPECT findings
Andin et al 2005^15^	Cardiac & neuropathology	Postmortem examination of patients in prospective, longitudinal study	Cardiovascular pathology in different types of vascular dementia; Relationship between cardiovascular & cerebrovascular disorders and type of vascular dementia	Prospective, longitudinal study of dementia	175	MI and hypertension in men are associated with small‐vessel dementia; coronary/aortic arteriosclerosis and MI more common in this group (than large‐vessel dementia/multi‐infarct dementia/hypoperfusive hypoxic–ischemic dementia)
Thore et al 2007^16^	Brain SVD	Descriptive neuropathology study	Determine an association between arteriolar tortuosity and leukoaraiosis	Autopsy cases	55	Arterial tortuosity in human cerebral white matter associates with coronary artery disease (the presence of vascular stenosis, either coronary or cerebrovascular, displayed the highest correlation with tortuosity (*P*=0.0582), almost reaching significance)
Park et al 2013^17^	Brain & heart SVD	Case report	Description of cardiac investigations in a patient with CADASIL	···	1	Myocardial SPECT showed reversible perfusion defects in the septum (possibly because of vascular disease in the septal perforators of the LAD)
Riverol et al 2015^18^	Brain & kidney SVD	Prospective cohort study	Determine whether SVD in the kidney can predict SVD in the brain	Baseline brain MRI and cystatin C levels and no history of dementia	735	Higher cystatin C levels are associated with more WMLs, lower GM volume, and poorer cognitive function 6 y later (is this because of common SVD process or does CKD lead to brain SVD?)
Yamamoto et al 2013^19^	Brain & systemic (skin) SVD	Descriptive immunochemistry and electron microscopy study	Examine the specific N3ECD accumulation in relation to GOM in the cerebral vasculature and brain parenchyma of CADASIL patients and compared findings with other non‐CADASIL hereditary and sporadic SVD of the brain	Patients with CADASIL, non‐CADASIL hereditary SVD and sporadic age‐related degenerative disease, and comparable‐age controls	75	N3ECD is predominantly localized within GOM deposits and the extensive distribution of N3ECD‐GOM complexes within meninges, arteries, arterioles, and brain capillaries of CADASIL patients suggests NOTCH3 fragments are major components of GOM deposits, which may be eliminated via perivascular routes

Publications are listed in chronological order. CADASIL indicates cerebral autosomal dominant arteriopathy with subcortical infarcts and leukoencephalopathy; CKD, chronic kidney disease; ECD,; GM, gray matter; GOM, granular osmiophilic material; LAD, left anterior descending artery; MI, myocardial infarction; N3ECD, NOTCH3 extracellular domain protein; SPECT, single‐photon emission computed tomography; SVD, small‐vessel disease; WMLs, white matter lesions.

## SVD in the Heart—Microvascular Angina

SVD in the heart was historically referred to as “Cardiac Syndrome X.” 3, 4 This term has been superseded by the more appropriate term microvascular angina (MVA). Symptoms in affected patients may be triggered by exertion, emotional stress, cold weather, the menstrual cycle, and menopause.^4^ Structural microvessel abnormalities, extravascular compressive forces, and abnormal coronary microvascular tone may be underpinning mechanisms leading to MVA.^4^ The diagnostic criteria for MVA have been recently proposed by the COVADIS (Coronary Vasomotion Disorders International Study) steering group (Table 2).^20^

**Table 2:** Diagnostic criteria for microvascular angina

Clinical criteria for suspecting MVA^a^
1 Symptoms of myocardial ischemia Effort and/or rest angina Angina equivalents (i.e., shortness of breath) 2 Absence of obstructive CAD (b = >50% diameter reduction or FFR N = ≤0.80) by Coronary CTA Invasive coronary angiography 3 Objective evidence of myocardial ischemia Ischemic ECG changes during an episode of chest pain Stress‐induced chest pain and/or ischemic ECG changes in the presence or absence of transient/reversible abnormal myocardial perfusion and/or wall motion abnormality 4 Evidence of impaired coronary microvascular function Impaired coronary flow reserve (cutoff values depending on methodology use between ≤2.0 and ≤2.5) Coronary microvascular spasm, defined as reproduction of symptoms, ischemic ECG shifts but no epicardial spasm during acetylcholine testing. Abnormal coronary microvascular resistance indices (e.g., IMR >25) Coronary slow flow phenomenon, defined as TIMI frame count >25.

CAD indicates coronary artery disease; CTA, computed tomographic angiography; FFR, fractional flow reserve; IMR, index of microcirculatory resistance; MVA, microvascular angina; TIMI, thrombolysis in myocardial infarction.

a Definitive MVA is only diagnosed if all 4 criteria are present for a diagnosis of MVA. Suspected MVA is diagnosed if symptoms of ischemia are present (criterion‐1) with no obstructive coronary artery disease (criterion‐2) but only (a) objective evidence of myocardial ischemia (criterion‐3), or (b) evidence of impaired coronary microvascular function (criterion‐4) alone.

Invasive coronary angiography is the key test for the diagnosis and treatment of coronary artery disease. However, since the diameter of coronary microvessels is typically <0.5 mm, they are too small to be resolved visually by the cardiologist. Therefore, angiography is primarily an investigation for large artery coronary disease, and only a subset (40–50%) of patients undergoing coronary angiography have obstructive disease identified.^21^, ^22^ Building on these studies, in the recent British Heart Foundation Coronary Microvascular Angina (CorMicA) trial, 185 of 391 (47%) patients with angina undergoing clinically‐indicated elective coronary angiography during a 12‐month period had no obstructive coronary artery disease when assessed using invasive coronary angiography and fractional flow reserve. SVD was identified in 134 (89%) of 151 patients who had invasive measurement of coronary vascular function. This result points to the high prevalence of SVD in patients with ischemia and no obstructive coronary disease (INOCA).^23^ The coronary slow‐flow phenomenon may be disclosed by angiography in a subset of patients with INOCA (Table 2). Patients with INOCA may have impaired health‐related quality of life comparable to that of patients with obstructive coronary artery disease,^22^ and prognosis may be affected.^3^, ^4^, ^22^, ^23^, ^24^ Compared with population‐matched controls, patients with INOCA have almost double the risk of death, myocardial infarction, and stroke over a 7.5‐year period.^25^

Establishing the correct diagnosis in the catheter laboratory is a patient‐centered approach. Since coronary angiography alone may be insufficient, SVD‐specific tests of coronary function should be considered in selected patients with INOCA. Invasive tests of microvascular function include a diagnostic guidewire to measure microvascular resistance directly and coronary flow reserve and/or intracoronary infusion of acetylcholine. In current cardiological practice, these are rarely used. The reasons are multifactorial. Lack of evidence from randomized controlled trials and inadequate education and training of physicians are relevant.^4^, ^26^, ^27^ The CorMicA trial^23^ has reduced this gap in evidence. For the first time, CorMicA provided proof‐of‐concept evidence that a management strategy involving routine use of coronary function tests at the time of invasive coronary angiography in patients with INOCA improved symptoms and quality of life, compared with standard management guided by coronary angiography. These results support a stratified medical approach involving specific tests for SVD with linked therapy. Overall, more education and research are needed to improve patient‐centered management.

## SVD of the Brain

Cerebral SVD (referred to in older literature as lipohyalinosis, Binswanger disease, subcortical leukoencephalopathy)^8^, ^25^ may manifest clinically as stroke (infarction) or a cognitive syndrome usually with executive dysfunction (because of subcortical white matter disruption or atrophy). Cerebral SVD is the primary cause of lacunar ischemic stroke, which represents ≈20% of all stroke. It appears to be the most common source of vascular contributions to cognitive impairment and dementia.^24^

Cerebral SVD encompasses a range of vascular pathologies including arteriolosclerosis, small‐vessel atheroma, and cerebral amyloid angiopathy as reviewed elsewhere.^9^, ^28^ Most prevalent is arteriolosclerosis, or “simple” SVD, which is a concentric hyaline thickening of deep penetrating small arteries (outer diameter <200 μm) with fibrosis of the vessel wall and depletion of vascular smooth muscle cells.^9^ This is detected as diffuse white matter hyperintensities on T2‐weighted MRI, associated with small focal ischemic lesions in subcortical areas, sometimes accompanied by microbleeds and, more rarely, deep intracerebral hemorrhage. While age and hypertension are strong risk factors, the molecular mechanisms in cerebral SVD are little known. Systematic review and meta‐analyses have suggested that white matter hyperintensities (indicative of underlying SVD) are associated with an increased risk of dementia (hazard ratio 1.9),^28^ whereas prospective population‐based data indicate that white matter hyperintensities are associated with 1.4‐fold increased risk of dementia.^9^, ^28^, ^29^

Vascular aging shares some pathophysiological features seen in hypertensive vascular disease. In the Atherosclerosis Risk in Communities prospective cohort study of 1827 participants age 45 to 64 years drawn from 4 regions in the United States,^30^ small lacunes defined as focal lesions hyperintense to gray matter on both proton density and T2‐weighted MRI were independently associated with age (per year: 1.12 [1.45–2.02]) and other vascular risk factors including hypertension (2.11 [1.50–2.97]), diabetes mellitus (1.34 [0.95–1.90]), and ever‐smoking (1.47 [1.06–2.03]).

## Possible Genetic Link Between Heart and Brain SVD

Cerebral autosomal dominant arteriopathy with subcortical infarcts and leukoencephalopathy (CADASIL) is a familial genetic form of cerebral SVD caused by mutations in the *NOTCH3* gene, which is expressed in vascular smooth muscle cells.^13^, ^15^, ^17^, ^31^ The pathological hallmark is deposition of granular osmophilic deposits in vascular smooth muscle cells. Radiologically and pathologically, it presents as a severe form of SVD with younger age of onset (usually before aged 50 years) and little hypertension dependence, relative to sporadic SVD. While CADASIL classically affects brain vessels, it has the potential for systemic changes in the microcirculation.^22^ Lesnik Oberstein et al^13^ first reported myocardial infarction as an incidental finding in a case series of patients with genetically confirmed CADASIL (Table 1). Using a core laboratory approach, they evaluated the ECGs of 15 unrelated families who had genotyping to rule‐in (n=41; mean age 46 years, 19 [46% men]) or rule‐out (n=22; mean age 40 years, 10 [45% men]) the *NOTCH3* mutation. They found ECG evidence of myocardial infarction in 10 of 41 mutation carriers while none of the 22 nonmutation carriers had any ECG evidence of myocardial infarction. Cardiac pathology in one deceased *NOTCH3* mutation carrier revealed minimal atherosclerosis in the coronary arteries, whereas microvessels exhibited irregular fibrosis and elastosis of the media. Park et al^17^ reported the case history of a 46‐year‐old woman who had CADASIL and who was hospitalized following a stroke. Brain MRI revealed severe ischemic white matter changes and multiple chronic infarcts. The ECG revealed poor R‐wave progression and subsequent stress‐rest ^99m^Tc‐tetrofosmin myocardial perfusion single photon emission computed tomography (SPECT) revealed reversible myocardial perfusion defects in the distribution of the left anterior descending coronary artery. Computed tomography coronary angiography excluded coronary artery disease, supporting a diagnosis of coronary SVD.

## Evidence Linking SVD in the Heart and Brain

In a clinical–pathological series of 175 cases described as “vascular dementia,” Andin et al^15^ found that cardiac pathologies were more prevalent in patients with pathological evidence of cerebrovascular SVD (characterized by subcortical lacunes) than in other vascular dementia groups (subtyped in their report as large‐vessel dementia, hypoperfusive, hypoxic–ischemic dementia, venous infarct dementia, and hemorrhagic dementia).^15^

In a population study of 735 cognitively normal adults 65 years and older, the Cardiovascular Health Study‐Cognition Study, Riverol et al^17^ demonstrated that renal glomerular dysfunction correlated with cerebral SVD. Serum cystatin C concentration, taken to represent renal SVD, was associated with lower neuropsychological tests scores, the presence of MRI‐identified brain infarcts, and the volume of white matter lesions.^18^ Age, waist circumference, hypertension, reduced physical activity, cigarette smoking, and C‐reactive protein were all multivariate correlates of cystatin C concentration.^18^ These results provide evidence that SVD may be a systemic disorder, potentially more pronounced in patients with multimorbidity, and that shared vascular risk factors are relevant.^32^

Three case series have found evidence of a high prevalence of abnormalities in cerebral blood flow in patients with cardiac SVD.^12^, ^14^, ^33^ Weidmann et al^33^ studied cerebral blood flow using technetium‐99m (Tc‐99m)‐*d,l*‐hexamethylpropyleneamineoxime SPECT in a consecutive series of 95 patients (mean age 55 years) with MVA. They found that 72 (76%) had an abnormal brain SPECT scan, with hypoperfusion lesions in the parietal lobes predominating. Sun et al^12^ reported similar findings (Table 1). Pai et al^14^ found that in a group of 30 patients with cardiac SVD, brain hypoperfusion lesions on technetium‐99m ethyl cysteinate dimer brain SPECT were common (21/30 patients) and positively associated with the presence and extent of abnormalities in myocardial perfusion as revealed by thallium‐201 myocardial perfusion SPECT. Brunelli et al^11^ studied cerebral blood flow using ^133^Xe inhalation and found no differences between 16 patients with MVA and 16 controls. None of these studies included repeated assessments over time, and more research into the natural history of heart and brain SVD seems justified. Taken together, these studies show that heart and brain hypoperfusion may co‐exist in patients with MVA, supporting the thesis of Sax et al^10^ of a multisystem SVD disorder.

Thore et al^16^ provided insights into the natural history of patho‐anatomical changes in brain small vessels with aging. They undertook a morphometric analysis of arteriolar tortuosity in human cerebral white matter of preterm, young, and aged subjects (age range 23 weeks postconception to 102 years). They used computerized morphometry to determine a vascular curl score (curvilinear length/straight length) in white matter arterioles in thick (100 μm) alkaline phosphatase–stained sections. They reported that the tortuosity score increased with age and showed borderline association with a history of IHD (*P*=0.058 for distribution).

In patients with acute subarachnoid hemorrhage, ECG changes including ST‐segment deviation and QT‐prolongation are common and an adverse prognostic factor.^34^ The extent and nature of the ECG changes correlate with vasospasm identified on cerebral arteriography.^35^ An increase in circulating troponin concentration is an adverse prognostic factor after subarachnoid hemorrhage.^36^ Although coronary tone has not been measured directly in patients with subarachnoid hemorrhage, these results implicate coronary vasospasm as a secondary process leading to myocardial ischemia in affected patients.

## Mechanisms of SVD Affecting the Heart and Brain

We hypothesize that SVD is a multisystem disorder with a common pathophysiological basis that differentially affects the heart and brain in some patients. The natural history is incompletely understood. Why some patients with MVA subsequently develop vascular cognitive impairment and others do not is an unanswered question. Potential underpinning mechanisms include premature vascular aging, clustering of vascular risk factors leading to an accelerated cardiovascular risk, and activation of the endothelin system.^9^, ^37^ Vascular fibrosis driven by the transforming growth factor β family of regulatory signaling proteins may also be causally relevant.^38^

## Premature Vascular Aging and Oxidative Stress

Vascular aging is associated with endothelial dysfunction,^38^ oxidative stress,^38^ increased blood vessel stiffness,^39^ impaired angiogenesis,^40^ rarefaction,^41^ and extracellular matrix changes.^42^ Degeneration and perivascular fibrosis in the microvasculature supplying cerebral periventricular white matter accumulate with age.^43^ Premature vascular aging may have a genetic component.^44^ Genetic susceptibility and interactions with environmental vascular factors (e.g., smoking, obesity, and lifestyle) may predispose to accelerated risk of clinical syndromes because of SVD in the heart and brain.

## Systemic Endothelial Dysfunction

In the CorMicA study,^45^ we tested the hypothesis that patients with INOCA also have functional abnormalities in peripheral small arteries. Using arterioles isolated from gluteal biopsies, we found that patients with microvascular angina and vasospastic angina had peripheral microvascular abnormalities characterized by reduced maximum relaxation following incubation with ACh (in keeping with endothelial dysfunction) and increased responses to vasoconstrictor stimuli. Our study provides evidence of associations between coronary microvascular dysfunction and SVD in other organs, such as the brain and kidney.

Endothelial activation is mechanistically implicated in SVD secondary to hypertension and associated with changes in cognitive performance over time.^46^ Circulating molecules that are mediators of endothelial dysfunction are implicated in the pathophysiology of SVD, leading to angina and cognitive decline. A systematic review and meta‐analysis of circulating markers of inflammation (C‐reactive protein, tumor necrosis factor‐α, interleukin‐6) and endothelial dysfunction (notably homocysteine and von Willebrand factor) disclosed associations with lacunes, but not circulating markers of coagulation and fibrinolysis.^47^

## Endothelin‐1

**Figure 3:**
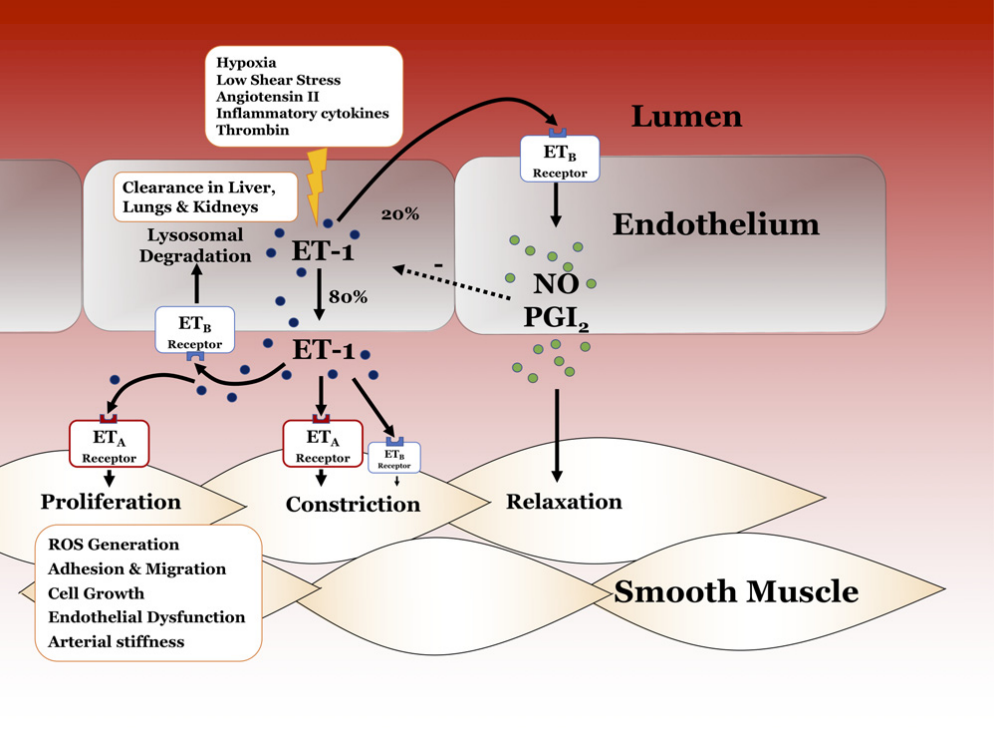
Endothelial function and harmony of the vascular endothelin system. There is complex homeostatic interplay between endothelial (dys)function and the effects of ET‐1 on vascular tone and atherogenic milieu. Endothelial dysfunction causes coronary and systemic (peripheral) microvascular disease and the underlying mechanisms involve dysregulation of the endothelin‐1 (ET‐1) system. EDN1 gene transcription in vascular endothelial cells produces pre‐pro ET‐1, which is cleaved to big ET‐1 and subsequently to ET‐1. Around 80% of ET‐1 secretion occurs abluminally, where it binds to ET_A_ and ET_B_ are G‐protein coupled receptors that are expressed on the vascular smooth muscle cell surface mediating constrictor and mitogenic effects. In healthy endothelial cells, luminal ET‐1 binds to and activates ET_B_ receptors, providing a crucial homeostatic role. Endothelial ET_B_ activation leads to eNOS activation and PGI2 and nitric oxide (NO) production. Endothelial dysfunction is associated with reductions in NO, prostacyclin, and endothelium‐derived hyperpolarizing factor and a preponderance of oxidants, ET‐1, and other vasoconstrictor and mitogenic substances within the vascular wall. ROS indicates reactive oxygen species.

Endothelin‐1 is implicated in the vascular pathophysiology of SVD in the heart and brain (Figure 3). Endothelin‐1 is a 21‐amino acid peptide that is released mainly by endothelial cells.^48^ Endothelin‐1 is a highly potent vasoconstrictor via its ET_A_ receptors expressed on vascular smooth muscle cells. In addition, this peptide has profibrotic, mitogenic, pro‐oxidant, pro‐inflammatory, and inotropic actions and regulates renal fluid and electrolyte homeostasis.^48^

Endothelin‐1 increases peripheral^49^ and coronary^50^ vascular tone via ET_A_‐activation.^51^ Endothelin‐1 contributes to coronary endothelial dysfunction,^50^ and its tonic inhibitory effect on myocardial perfusion, as revealed by positron emission tomography, is related to the presence and extent of risk factors for atherosclerosis.^52^ Kaski et al^53^ observed that in patients with MVA, circulating endothelin‐1 concentrations were increased and associated with a shorter time to onset of angina during exercise. In subsequent studies,^54^ they showed that increased endothelin‐1 activity is associated with reduced coronary flow responses, notably in women. Using rest/dipyridamole positron emission tomography with Rb‐82 for the assessment of IHD, Johnson et al^55^ identified an abnormal pattern of diffuse heterogeneous myocardial perfusion that was associated with coronary SVD. They observed that in patients with heterogeneous reductions in resting myocardial perfusion (consistent with coronary SVD), treatment with the ET_A_ antagonist, darusentan, improved myocardial perfusion, and increased the homogeneity of the perfusion pattern. They concluded that in patients with coronary SVD, endothelin‐1 caused regional reductions in myocardial perfusion and that these abnormalities could be improved by ET_A_ receptor blockade. In a randomized placebo‐controlled trial of an oral ET_A_ antagonist (atrasentan, 10 mg PO daily) administered for 6 months in 47 patients with coronary microvascular disease, Reriani et al^56^ observed that chronic ET_A_ antagonist therapy improved microvascular coronary endothelial function. This change was accompanied by greater reductions in mean arterial blood pressure and plasma glucose.^56^ Recent genetic fine mapping linked the endothelin gene, *EDN1*, to multiple cardiovascular disease states, including coronary heart disease, coronary calcification, migraine headache, cervical artery dissection, fibromuscular dysplasia, and hypertension.^57^ In the CorMicA study,^45^ peripheral arterioles isolated from patients with INOCA had enhanced vasoconstriction in response to ET‐1 and the thromboxane agonist U46619 compared with vasoconstrictor responses from control subjects. The results support the provocative concept that patients with INOCA are at risk of developing generalized SVD.

## Future Directions

Many of the current studies have limitations, such as their cross‐sectional design and lack of longitudinal follow‐up. Age‐related changes may be a confounding factor in the associations between SVD in the heart and brain. Longitudinal studies of the pathological changes and risk factors with appropriate controls would help in better understanding the natural history of these conditions. For example, are patients with microvascular disease in the heart indeed more likely to develop vascular cognitive impairment? Potential therapeutic targets may emerge and the effects of endothelin‐1 receptor antagonists in cerebral SVD would be of interest.

## Advances for Diagnosis, Treatment, and Epidemiology of SVD in the Heart and Brain

The positive results from the CorMicA study should be investigated further for external validity in a multicenter trial. Nuclear imaging with SPECT and positron emission tomography and cardiovascular MRI are highly informative for investigating ischemia in the heart and brain. Advances in cardiovascular MRI now enable quantitative measurements of myocardial blood flow (mL/min per g tissue) with pixel‐level resolution in near real‐time,^58^ which holds promise to be diagnostically useful for patients with INOCA with potentially a combinatory approach with advanced cardiovascular MRI in the heart and brain.

Advances in brain imaging to quantify SVD include diffusion imaging at 3.0 T, susceptibility‐weighted MRI (to detect cerebral microbleeds), T1‐weighted MRI (lacunes), fluid‐attenuated inversion recovery MRI (white matter hyperintensities), diffusion tensor imaging (white matter integrity), subcortical atrophy (3D‐T1‐weighted imaging), and brain arterial spin labeling to map regional cerebral blood flow with CO_2_ challenge to quantify cerebrovascular reactivity. Recent advances in brain imaging have evidenced the clinical significance of microbleeds, which are a biomarker for some manifestations of SVD. MRI at 7.0 T offers a number of novel insights into the arterial and parenchymal lesions associated with SVD.^59^ MRI at 7.0 T visualizes perforating arteries, cerebral micro‐infarcts, and lesions in the arterial walls. Future research using 7.0 T MRI of the brain in patients with cardiac SVD seems warranted.

## Advances in Therapy

Preventive measures for SVD in at‐risk or affected individuals currently focus on modification of vascular risk factors, notably hypertension, obesity, and smoking. Lifestyle interventions, notably through regular aerobic exercise, are recommended.^3^, ^4^, ^27^ There are no targeted specific disease‐modifying therapies for SVD in the heart or brain, presenting a major opportunity for research and potential therapeutic intervention.^3^, ^5^, ^6^

## Endothelin‐1 Receptor Antagonists

Endothelin‐1 receptor antagonists are an established treatment for microvascular disease in the lung; for example, they are a drug of choice for pulmonary arterial hypertension. Although they were thought to have renoprotective effects, the SONAR trial (ClinicalTrials.gov Identifier: NCT01858532; atrasentan phase 3 trial, diabetic nephropathy) closed early (Q4.2017) because of a lack of primary end‐point events in the study population. Two small randomized trials of an endothelin‐1 receptor antagonist in MVA^55^, ^56^ had favorable results, but these compounds are not available following “negative” phase 2/3 trials in oncology and hypertension. There were no safety concerns.

## Rho Kinase Inhibitors

The RhoA/Rho kinase system plays an important role in vasoconstriction. RhoA/Rho kinase inhibitors have therapeutic potential for patients with MVA. A study of the effects of SAR407899 on coronary vasomotor function using coronary flow reserve in patients with MVA (NCT03236311) was halted early because of slow enrollment. This outcome reflects the need for future trials to adopt eligibility criteria and methods of assessment that facilitate enrollment.

## Conclusion

Our review provides evidence that abnormalities in cerebral blood flow are common in patients with MVA and that SVD can be considered a multisystem disorder. Vascular risk factors alone cannot explain INOCA because many patients with MVA lack risk factors for vascular disease. Key gaps in knowledge include (1) the natural history and prognosis of multisystem SVD; (2) causal genetic variants; (3) underlying molecular mechanisms; (4) optimal diagnostic methods for SVD in heart, brain, and other organs; and (5) preventive and/or disease‐modifying therapy (pharmacological and nonpharmacological).

## Sources of Funding

This work was supported by funding from the British Heart Foundation (BHF) (RE/18/6/6134217; PG/17/25/32884; FS/17/26/32744) and Alzheimer's Society (UK) (Project Ref 20140901).

## Disclosures

C.B. is employed by the University of Glasgow, which holds consultancy and research agreements with companies that have commercial interests in the diagnosis and treatment of angina. The companies include Abbott Vascular, AstraZeneca, Boehringer Ingelheim, HeartFlow, Menarini Pharmaceuticals, Philips, and Siemens Healthcare. These companies had no involvement in this article. A.H.H. has funding from Alzheimer's Society (UK) and Alzheimer's Drug Discovery Foundation (Project Ref 20140901) to carry out a clinical trial of the PDE5 inhibitor drug tadalafil for possible use in SVD and A.C.P. is part of the study team. A.H.H. has received honoraria from Eli Lilly and from the National Institute on Aging. A.H.H. and R.M.T. are both members of the Dementia Platform UK vascular experimental medicine study group. The remaining authors have no disclosures to report.

## Supporting information


**Table S1.** Relevant Articles Identified in Literature Review Not Relating to SVD in the Heart and Brain
