# Supplementary material for: Small‐Vessel Disease in the Heart and Brain: Current Knowledge, Unmet Therapeutic Need, and Future Directions
Source: J Am Heart Assoc. 2019 Feb 2;8(3):e011104. doi: 10.1161/JAHA.118.011104 (PMC6405580; doi:10.1161/JAHA.118.011104)
Supplement: Supplementary file 1 — Table S1. Relevant Articles Identified in Literature Review Not Relating to SVD in the Heart and Brain [file JAH3-8-e011104-s001.pdf]

SUPPLEMENTAL MATERIAL

Table S1. Relevant papers identified in literature review not relating to SVD in the heart and brain.

Authors/Date	SVD component	Design	Objective	Focus	Time frame	Sample size	Relevant findings
Gerdes et al 2006[1]	Brain SVD	Prospective cohort study	Determine if WML is associated with future extra-cerebral ischaemic events	Patients with recent CVA/MI/PAD	1992-1994	230	Cerebral WML (especially PVL) are associated with ischaemic events (IS & MI) – possible ischaemia in an arterial borderzone, hypoperfusion caused by large vessel disease, diminished cerebral vasomotor reactivity, or SVD developing parallel to systemic large vessel disease
van Elderen et al 2010[2]	Brain SVD	Prospective cohort study	Determine (with MRI) if aortic stiffness (reflected by aortic pulse wave velocity) is associated with LV function/mass and cerebral SVD	Patients with Type 1 diabetes	2008-2009	86	Aortic stiffness is associated with LV systolic function and cerebral WMH in patients with Type 1 diabetes (independent of hypertension)
Conijn et al 2011[3]	Brain SVD	Prospective cohort study	Determine if WML & LI (markers of SVD) increase risk of vascular and nonvascular deaths	Patients with atherosclerotic disease and WML/LI on brain MRI	2001-2005	1228	WML & LI increase risk of all-cause and vascular deaths; LI increases risk of nonvascular deaths too; WML & LI are not associated with "ischaemic cardiac complications"
Poels et al 2012[4]	Brain SVD	Population-based cohort study	Determine if arterial stiffness is associated with cerebral SVD (WML, LI, CMB)	Elderly patients in the Netherlands	1990s	1460	Arterial stiffness is associated with a larger volume of WML (especially in patients with uncontrolled hypertension) but not LI or CMB
Shimizu et al 2014[5]	Brain SVD	Prospective cohort study	Determine if WML is associated with LV diastolic dysfunction	Patients between 65 & 75 with normal LVSF and no history of HF/IHD/AF/CVA/dementia	2010-2012	75	WML is associated with LV diastolic dysfunction (but no causal relationship can be determined)
Harbaoui et al 2015[6]	Brain SVD	Prospective cohort study	Determine contributions of BP, aortic stiffness and SVD on coronary events,	Patients with hypertension	1969-1976	1031	Atherosclerosis score & pulse pressure are associated with coronary events; Mean BP is associated with cerebrovascular and renal-related

			cerebrovascular events and renal-related events				deaths; Retinopathy is associated with cerebrovascular-related deaths
Kamel et al 2015[7]	Brain SVD	Prospective cohort study	Determine if ECG LA abnormality (and therefore LA disease is associated with stroke (both cortical infarcts and leukoaraiosis)	Patients with a baseline brain MRI and no history of AF/CVA	1989-1993	3129	ECG LA abnormality is associated with vascular brain injury, especially non-lacunar infarcts
Bang et al 2016[8]	Brain SVD	Prospective cohort study	Determine risk factors of intracranial microangiopathy & macroangiopathy	Patients with MCA infarcts in tertiary stroke centre	2008-2012	714	Mild-moderate renal dysfunction associated with both microangiopathy & macroangiopathy, possibly due to endothelial dysfunction
Leung et al 2017[9]	Brain SVD	Prospective cohort study	Determine if BP and HR are associated with with incident brain infarcts and worsening leukoaraiosis	Patients with a baseline brain MRI and no history of CVA	1989-1993	878	Elevated SBP is associated with increased risk of covert brain infarction and elevated DBP is associated with increased risk for worsening leukoaraiosis

SVD: small vessel disease, MCA: middle cerebral artery, WML: white matter lesion, LI: lacunar infarct, MRI: magnetic resonance imaging scan, CVA: cerebrovascular accident, MI: myocardial infarction, PAD: peripheral arterial disease, PVL: periventricular leukomalacia, IS: ischaemic stroke, BP: blood pressure, ECG: electrocardiogram/electrocardiograph, LA: left atrial, AF: atrial fibrillation, HR: heart rate, SBP: systolic blood pressure, DBP: diastolic blood pressure, CMB: cerebral microbleeds, LV: left ventricular, LVSF: left ventricular systolic function, HF: heart failure; IHD: ischaemic heart disease, WMH: white matter hyperintensities.

Supplemental References:

1. Gerdes VE, Kwa VI, ten Cate H, Brandjes DP, Buller HR, Stam J. Cerebral white matter lesions predict both ischemic strokes and myocardial infarctions in patients with established atherosclerotic disease. *Atheroscler.*2006;186:166-72.
2. van Elderen SG, Brandts A, Westenberg JJ, van der Grond J, Tamsma JT, van Buchem MA, Romijn JA, Kroft LJ, Smit JW, de Roos A. Aortic stiffness is associated with cardiac function and cerebral small vessel disease in patients with type 1 diabetes mellitus: assessment by magnetic resonance imaging. *Eur Radiol.*2010;20:1132-8.
3. Conijn MM, Kloppenborg RP, Algra A, Mali WP, Kappelle LJ, Vincken KL, van der Graaf Y, Geerlings MI; SMART Study Group. Cerebral small vessel disease and risk of death, ischemic stroke, and cardiac complications in patients with atherosclerotic disease: the Second Manifestations of ARTerial disease-Magnetic Resonance (SMART-MR) study. *Stroke.*2011;42:3105-9.
4. Poels MM, Zaccai K, Verwoert GC, Vernooij MW, Hofman A, van der Lugt A, Witteman JC, Breteler MM, Mattace-Raso FU, Ikram MA. Arterial stiffness and cerebral small vessel disease: the Rotterdam Scan Study. *Stroke.*2012;43:2637-42.
5. Shimizu A, Sakurai T, Mitsui T, Miyagi M, Nomoto K, Kokubo M, Bando YK, Murohara T, Toba K. Left ventricular diastolic dysfunction is associated with cerebral white matter lesions (leukoaraiosis) in elderly patients without ischemic heart disease and stroke. *Geriatr Gerontol Int.*2014;14 Suppl 2:71-6.
6. Harbaoui B, Courand PY, Milon H, Fauvel JP, Khettab F, Mechtouff L, Cassar E, Girerd N, Lantelme P. Association of various blood pressure variables and vascular phenotypes

with coronary, stroke and renal deaths: Potential implications for prevention. *Atheroscler*.2015;243:161-8.

7. Kamel H, Bartz TM, Longstreth WT, Jr., Okin PM, Thacker EL, Patton KK, Stein PK, Gottesman RF, Heckbert SR, Kronmal RA, Elkind MS, Soliman EZ. Association between left atrial abnormality on ECG and vascular brain injury on MRI in the Cardiovascular Health Study. *Stroke*.2015;46:711-6.

8. Bang OY, Chung JW, Ryoo S, Moon GJ, Kim GM, Chung CS, Lee KH. Brain microangiopathy and macroangiopathy share common risk factors and biomarkers. *Atheroscler*.2016;246:71-7.

9. Leung LY, Bartz TM, Rice K, Floyd J, Psaty B, Gutierrez J, Longstreth WT Jr, Mukamal KJ. Blood Pressure and Heart Rate Measures Associated With Increased Risk of Covert Brain Infarction and Worsening Leukoaraiosis in Older Adults. *Arterioscler Thromb Vascular Biol*.2017;37:1579-86.
